# Changing use of traditional healthcare amongst those dying of HIV related disease and TB in rural South Africa from 2003 – 2011: a retrospective cohort study

**DOI:** 10.1186/1472-6882-14-504

**Published:** 2014-12-17

**Authors:** Paul Mee, Ryan G Wagner, Francesc Xavier Gómez-Olivé, Chodziwadziwa Kabudula, Kathleen Kahn, Sangeetha Madhavan, Mark Collinson, Peter Byass, Stephen M Tollman

**Affiliations:** Medical Research Council/Wits University Rural Public Health and Health Transitions Research Unit (Agincourt), School of Public Health, Faculty of Health Sciences, University of the Witwatersrand, Johannesburg, South Africa; Umeå Centre for Global Health Research, Division of Epidemiology and Global Health, Department of Public Health and Clinical Medicine, Umeå University, Umeå, Sweden; International Network for the Demographic Evaluation of Populations and Their Health (INDEPTH) Network, Accra, Ghana; Department of African-American Studies, University of Maryland-College Park, College Park, MD USA; Department of Global Health and Development, Faculty of Public Health and Policy, London School of Hygiene and Tropical Medicine, London, UK; WHO Collaborating Centre for Verbal Autopsy, Umeå, Centre for Global Health Research, Department of Public Health and Clinical Medicine, Umeå University, Umeå, Sweden

**Keywords:** Traditional medical practitioner, Traditional medicine, Antiretroviral therapy, HIV, AIDS, Mortality, Tuberculosis, Demographic surveillance system, South Africa, Sub-Saharan Africa, Risk factor

## Abstract

**Background:**

In 2011 there were 5.5 million HIV infected people in South Africa and 71% of those requiring antiretroviral therapy (ART) received it. The effective integration of traditional medical practitioners and biomedical providers in HIV prevention and care has been demonstrated. However concerns remain that the use of traditional treatments for HIV-related disease may lead to pharmacokinetic interactions between herbal remedies and ART drugs and delay ART initiation. Here we analyse the changing prevalence and determinants of traditional healthcare use amongst those dying of HIV-related disease, pulmonary tuberculosis and other causes in a rural South African community between 2003 and 2011. ART was made available in this area in the latter part of this period.

**Methods:**

Data was collected during household visits and verbal autopsy interviews. InterVA-4 was used to assign causes of death. Spatial analyses of the distribution of traditional healthcare use were performed. Logistic regression models were developed to test associations of determinants with traditional healthcare use.

**Results:**

There were 5929 deaths in the study population of which 47.7% were caused by HIV-related disease or pulmonary tuberculosis (HIV/AIDS and TB). Traditional healthcare use declined for all deaths, with higher levels throughout for those dying of HIV/AIDS and TB than for those dying of other causes. In 2003-2005, sole use of biomedical treatment was reported for 18.2% of HIV/AIDS and TB deaths and 27.2% of other deaths, by 2008–2011 the figures were 49.9% and 45.3% respectively. In bivariate analyses, higher traditional healthcare use was associated with Mozambican origin, lower education levels, death in 2003–2005 compared to the later time periods, longer illness duration and moderate increases in prior household mortality. In the multivariate model only country of origin, time period and illness duration remained associated.

**Conclusions:**

There were large decreases in reported traditional healthcare use and increases in the sole use of biomedical treatment amongst those dying of HIV/AIDS and TB. No associations between socio-economic position, age or gender and the likelihood of traditional healthcare use were seen. Further qualitative and quantitative studies are needed to assess whether these figures reflect trends in healthcare use amongst the entire population and the reasons for the temporal changes identified.

**Electronic supplementary material:**

The online version of this article (doi:10.1186/1472-6882-14-504) contains supplementary material, which is available to authorized users.

## Background

Traditional Medical Practitioners (TMPs) are an important component of the available healthcare resources in South Africa. They often provide services with greater cultural acceptability and geographical accessibility than their biomedical counterparts [1]. The World Health Organization (WHO) defines traditional medicine as referring to ‘*health practices, approaches, knowledge and beliefs incorporating plant, animal and mineral based medicines, spiritual therapies, manual techniques and exercises, applied singularly or in combination to treat, diagnose and prevent illnesses or maintain well-being*’ [2]. In South Africa these practitioners include herbalists (izinyanga/inyanga or amaxhwele), diviners (izangoma/sangoma or amagqirha), traditional surgeons and traditional birth attendants [3]. In the treatment of HIV-related conditions, the holistic emphasis of the care provided by TMPs addressing psychosocial and physical aspects of disease has the potential to complement biomedical treatments and has led to efforts to integrate them with biomedical practitioners in the diagnosis and treatment of those with HIV [1, 4, 5]. They have been reported to also have an important role to play in palliative care for those with HIV/AIDS [6, 7]. Evidence from studies conducted prior to 1994 indicated that when traditional healers are properly trained they can make a useful contribution to HIV prevention, care and support [5]. More recently, notable successes in linking traditional healers with those in other health sectors have been reported from projects in Tanzania, Uganda, Zambia, Malawi and other sub-Saharan African countries , although there have been a lack of systematic evaluations of these interventions [5].

As TMPs share the same socio-cultural beliefs as their communities, they have the potential to be effective communicators of health information [8]. It has been estimated that 70 - 80% of South Africans consult TMPs [9] although there is evidence for a decline in use in recent years [3]. Amongst the possible explanations for this reduction are that there has been an increased belief in the efficacy of biomedical treatment [3] and that some churches have discouraged their members from seeking traditional medical care [10, 11].

In 2011 the prevalence of HIV amongst males and females in South Africa aged 15 – 49 was estimated to be 17.6% with a total of 5.5 million people infected by HIV [12]. In the early stages of the epidemic there was scepticism on the part of the South African government about the link between the HIV virus and the developing AIDS pandemic and also doubts about the efficacy of antiretroviral therapy (ART) for treating the condition [13]. At the same time the use of herbal medicines to treat those infected with HIV was promoted by the South African Ministry of Health [14]. ART was made available to patients in the South African public health system in 2003 but there were delays in the national roll-out particularly in rural areas [15]. Estimates by WHO/UNAIDS and UNICEF indicate that ART coverage in South Africa in 2011 was 71% [16]. The availability of ART in South Africa has led to significant decreases in HIV-related mortality [17, 18].

There is evidence of pharmacokinetic interactions between certain herbal treatments and ART drugs [14, 19, 20]. Some studies have shown that patients using herbal treatments in conjunction with ART are more likely to take gaps in treatment or reduce their level of adherence [21, 22]. There are also concerns that those using herbal remedies to treat HIV-related symptoms prior to starting ART may delay the initiation of treatment [23]. An association between the use of traditional medicine and delayed initiation of TB treatment was identified in a rural hospital in South Africa in 2006 [24].

Seeking treatment from TMPs can also place a significant financial burden on the patient and their family at a time when household resources are likely to be under considerable strain [25]. Babb et al. reporting on individuals attending a workplace ART clinic [26] and Case et al. who interviewed family members of the deceased [27], both noted the high cost of a course of treatment from a herbalist. In the latter study carried out in KwaZulu-Natal in South Africa between 2003 and 2004 the average cost of treatment by a traditional herbalist was found to be R433 (approximately 40 USD at the time) which was reported to be more than 4 times the median monthly per-capita income [27].

Between 2002 and 2003 a study of healthcare related beliefs was carried out in the Agincourt sub-district of Bushbuckridge in South Africa [28]. This concluded that a healthcare marketplace existed in which individuals selected from the available treatment providers based on their perception of the relative efficacies of the treatments offered. This study also found that late stage HIV-disease related symptoms were often interpreted as evidence of bewitchment and hence appropriate for treatment by traditional healers [28, 29]. This was in contrast to tuberculosis, for which the community had evidence of the effectiveness of the available short-course drug regimen [28]. Also prior to the ART roll-out some traditional healers in the Agincourt sub-district promoted treatments that were claimed to cure HIV [29]. A study of verbal autopsy data collected in the same area as the current study in 2006 [30] showed that witchcraft remained a common explanation for death at that time and that traditional remedies either used alone or in conjunction with biomedical treatments were commonly used to treat those perceived as bewitched.

With the availability of ART, patterns of medical pluralism became evident. A study of HIV clinic attendees carried out in the Agincourt sub-district between 2006 and 2007 identified that 5 out of 32 participants used informal sources of healthcare, which included traditional healers, as their first interaction with the medical system [23]. This study also found that the majority of the population remained in the public system after ART initiation whilst a minority switched between formal, informal and private sources of care over time.

A 2005 study in Gabon [31] and a 2008-2011 study in Kampala, Uganda [32] found that patients who had reported having received treatment from traditional healers or other informal sources had lower CD4 counts at treatment initiation. However no such association was found in another Ugandan study which recruited patients between 2005 and 2009 [33].

A 2007-2009 study carried out in KwaZulu-Natal, South Africa showed a significant decline in the parallel use of traditional medicines and ART from 36% at the initiation of treatment to 8% at 6 months, 4.1% at 12 months and 0.6% after 2 years treatment [21].

Previous South African studies have identified determinants associated with the use of traditional healthcare amongst HIV infected individuals. A 2003-2004 study in Northern KwaZulu-Natal [27] reported an increase in the use of traditional healers for females compared to males, those with higher levels of education, lower numbers of household assets, of a younger age and those who had been ill for a longer period. In a study of 618 treatment naïve patients attending an HIV clinic in KwaZulu-Natal between 2007 and 2008 [34] it was found that being on a disability grant and having fewer clinic visits increased the likelihood of a reported use of herbal treatments. A small pilot study in 2012 carried out in two South African sites, one in rural KwaZulu-Natal, the other an urban area of the Western Cape [35] found that having a rural dwelling, female gender, older age, a lack of formal education, not being married, having employment and having been HIV positive for less than 5 years were all predictors of traditional medicine use amongst people living with AIDS. The small sample size of this study however meant that none of the associations were statistically significant. A cross-sectional study of 334 patients at an HIV clinic in Western Uganda in 2010 [36] found that longer periods of time since ART initiation, greater numbers of ART side effects, lower levels of knowledge about ART and poorer self-perceived health status were all independently associated with concomitant herbal medicine and ART use, however no evidence for decreased ART adherence amongst the patients taking traditional medicines was found. In contrast to this a 2008 study in Uganda found that patients who had been on ART for less than 4 years were more likely to use traditional herbal medicines (THM) [37]. In addition this study found higher levels of use of THM amongst those experiencing side effects of ART, those less than 39 years old and those with ART adherence levels less than 95%.

A study [38] surveying traditional healthcare use in Ghana identified an association between rising income and an increased use of biomedical treatment whereas decreasing income was associated with a greater use of traditional medicine.

In the present study we analysed differences in the prevalence of the reported use of traditional and biomedical healthcare between those dying of HIV-related disease and pulmonary tuberculosis and those dying of other conditions and how the usage changed over time. The study used data collected between 2003 and 2011, the period during which ART became available in the area. We assessed determinants of traditional medicine use amongst those dying of HIV/AIDS related diseases and pulmonary tuberculosis.

## Methods

### Study setting

The data was collected from village communities in the Agincourt sub-district of Bushbuckridge Municipality in Mpumalanga province in north-eastern South Africa. The MRC/Wits Agincourt research unit has been running a Health and Socio-Demographic Surveillance System (HDSS) monitoring vital events in this area since 1992 [39]. The area is predominantly rural with nearby peri-urban settlements and has high levels of unemployment and poverty and low levels of educational attainment. Approximately one-third of the population are of Mozambican descent, the majority of these having been displaced by the Mozambican civil war (1977–1992) [40]. Voluntary counselling and testing (VCT) for HIV has been available at public health clinics in the study area since 1999 [41]. In the public sector ART has been available from two public hospitals serving the site since 2004 and from three local clinics serving the site since 2007. Initially there was only capacity to enrol limited numbers at these clinics but capacity has subsequently improved [42, 43].

Between August 2010 and May 2011 an HIV prevalence survey was conducted in the study site. It showed an overall HIV prevalence of 19.4% (10.6% for males and 23.9% for females). An analysis by age group showed a peak prevalence of over 45% in both males and females aged between 35–39 years. The HIV prevalence in those of South African origin was lower than for those from Mozambique [44].

The study population consists of adults (aged over 18) who died in the study site between 2003 and 2011 for whom healthcare utilisation data was available.

### Data collection

Individual and household level data were collected from the most knowledgeable available household member by trained fieldworkers during the annual census update visit. In households where a death was reported a visit was carried out by a specially trained fieldworker during which a verbal autopsy (VA) interview was completed with the person responsible for the care of the deceased during their terminal illness or the closest available proxy. This was done in order to determine the symptoms and circumstances associated with the illness leading to death [39, 45]. A health care utilisation (HCU) module was introduced to the Agincourt HDSS VA instrument in 2003 and included questions on the deceased’s use of traditional treatments. The HCU module questionnaire used with the Agincourt HDSS VA questionnaire is included as Additional file 1 in the additional materials.

### Data analysis

Cause of death (CoD) was assigned using the InterVA-4 model, which estimates the likelihood of a particular CoD based on signs and symptoms reported by the VA respondent [46, 47]. InterVA-4 is fully aligned to the questions and CoD assignments in the WHO-2012 VA instrument [48]. A previous study has shown a close correlation between clinician’s assessments and InterVA-4 assignments for HIV-disease related deaths [49]. The input variables for the InterVA-4 model were derived from the Agincourt VA questionnaire either by direct matching or identifying indicator specific words or phrases in narrative fields.

Data from 2009 [50] indicated that approximately 70% of those infected with pulmonary tuberculosis (TB) were co-infected with HIV in South Africa. A very high level of HIV co-infection amongst those dying of pulmonary TB has also been reported in a survey of various sites across sub-Saharan Africa [51]. Due to this high level of co-morbidity deaths due to HIV-related disease and pulmonary tuberculosis were joined in a single category, HIV/AIDS and TB. All other deaths were assigned to the category ‘Other Causes’ expect for those deemed to be accidental or self-inflicted, which were defined as ‘External’.

When the respondent reported the use of a TMP or self administration of herbal remedies the case was assigned as use of traditional healthcare (TH). The time period was broken down into 3 year categories: 2003–2005 (the period before ART became available in the study area); 2006–2008 (the period during which limited numbers of people were accessing ART in some local clinics) and 2009–2011 (when ART was made accessible in the public healthcare system in the area).

A map was produced using the ArcGIS software [52], showing the location of residence and whether TH use was reported for each deceased person. Spatial coordinates were randomly adjusted by between -0.5 km and 0.5 km in X and Y directions to protect anonymity. This was carried out in order to allow a visual investigation of whether there was evidence for higher levels of use of traditional healthcare in some areas of the study site than others.

The variables examined in this study were selected on the basis of either reports of significant associations with the use of traditional medicine in previous studies [27, 35] or *a priori* assumptions about their significance. A deceased individual was categorised as either a permanent resident if they dwelt in the study site for more than 6 months of the year preceding the household follow-up visit prior to their death or a temporary migrant if they dwelt in the site for 6 months or less. Individuals were assigned Mozambican origin if they either in-migrated from Mozambique or had a father classified Mozambican. Other than those from Mozambique all other residents reported South Africa as their country of origin. Individual educational attainment was calculated from the number of years of full time education successfully completed. Household socio-economic position (SEP) is a measure of asset ownership. It was derived from data on features such as the type of household building materials used, access to water and fuel and the ownership of appliances, livestock and transport. From these data 5 sub-indicators were constructed within which each asset variable was equally weighted. The sub-indicators were combined and standardised leading to the derivation of an absolute SEP score [53, 54]. Using the SEP score, households were ordered into asset status quintiles with quintile 1 representing the households with the least assets and quintile 5 those with the most. The duration of the final illness is based on the recollection of the VA respondents. The prior mortality rate at an individual’s dwelling was included in order to obtain a measure of the mortality experience of those who were co-resident with the deceased. This was calculated by dividing the total number of deaths in the dwelling by the total number of person years for all residents, accumulated over the period that the deceased lived there. Using the ArcGIS Network Analyst module, the road distances between each dwelling and the closest health centre were calculated.

Data were analysed using Stata version 10.0 [55]. Cross-tabulations with Chi-squared calculations for categorical variables and t-tests for continuous variables were used to assess the significance of associations between variables and the impact of missing data. Two-sample tests of proportions were used to calculate 95% confidence intervals (CI) for percentages and to compare differences between percentages for different causes of death in the same time period.

Logistic regression models were developed for those assigned to have died of HIV/AIDS and TB using an initial series of bivariate models with an outcome of 1 if the deceased used traditional or herbal treatments at any time during the final illness. A weighting term scaled from 0.4 to 1.0 was introduced in the regression to account for the likelihood of the cause of death as assigned by the InterVA-4 model. Continuous variables which showed an independent association with the outcome (p < 0.10) and categorical variables where there was an association in at least one category (p < 0.10) were included in the multivariate model. In order to understand whether the associations with TH use changed over time, interaction terms were introduced between each variable in the multivariate model and time period. Significant interactions were identified if the p-value in one category of the interaction term was less than 0.1 and the introduction of the interaction term improved the predictive power of the model as evidenced by an increase in the log likelihood.

### Ethics

Ethical clearance for the collection of data used in this study was given by the University of the Witwatersrand Human Research Ethics Committee (Medical) clearance certificates M960720 and M110138 ‘Investigating and Responding to Changes in the Health and Population Dynamics of Rural South Africans’. Verbal consent was obtained from each respondent prior to every follow-up survey.

## Results

**Figure 1:**
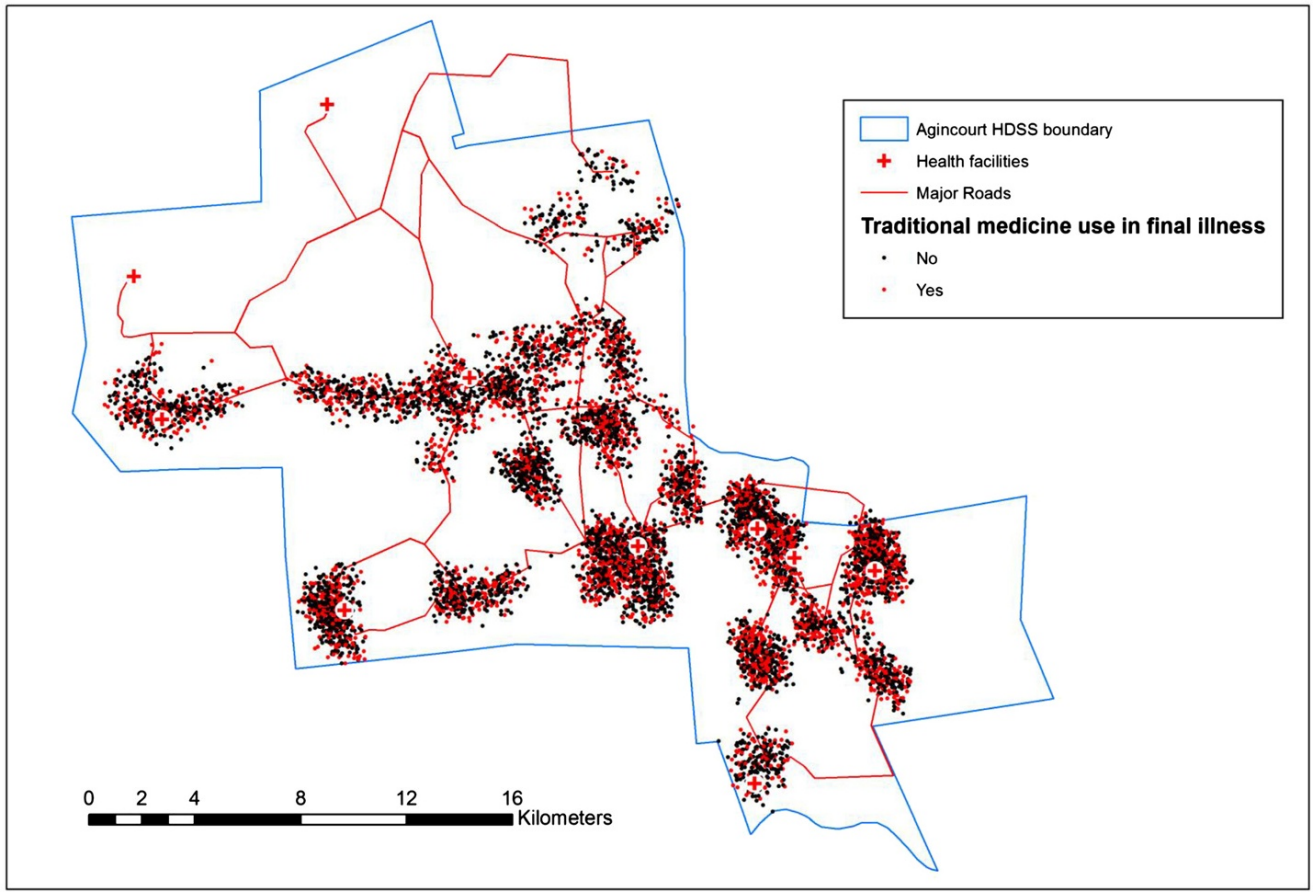
**Short title - Residential locations and traditional healthcare use for those dying between 2003 and 2011.** Detailed legend - This map shows the geographical distribution of the home location for those using traditional or herbal medicines during their final illness. To preserve anonymity the geographical coordinates are randomly shifted in the range +/- 0.5 km in the X and Y directions.

There were 6392 deaths recorded for those aged 18 years and over between 2003 and 2011. Health care utilisation (HCU) data was available for 5929 individuals (92.8%), who constituted the study population (Additional file 2: Table S1). The geographical distribution of the households and TH use status of those who died is shown in Figure 1. There was no indication from this of variations in likelihood of TH use amongst the deceased across the site. The characteristics of the study population are shown in Table 1. There were 2833 deaths attributable to HIV/AIDS and TB, 47.8% of the total.

**Table 1:** **Characteristics of the study population** ^**1**^

	Characteristic	n	%	(95% CI)
	Total	5929	100.0	
**Gender**	Female	2831	47.7	(46.0 - 49.5)
	Male	3098	52.3	(50.4 - 54.1)
**Age at death (years)**	18 to 49	3283	55.4	(53.5 - 57.3)
	50 to 65	1167	19.7	(18.6 - 20.8)
	>65	1479	24.9	(23.7 - 26.2)
**Country of origin** ^**2**^	Mozambique	1823	30.7	(29.3 - 32.2)
	South Africa	4101	69.2	(67.1 - 71.3)
	Unknown	5	0.1	(0.0 - 0.2)
**Year of death**	2003 to 2005	1781	30.0	(28.6 - 31.4)
	2006 to 2008	2115	35.7	(34.2 - 37.2)
	2009 to 2011	2033	34.3	(32.8 - 35.8)
**Residence status**	Temporary	1629	27.5	(26.1 - 28.8)
	Permanent	4166	70.3	(68.1 - 72.4)
	Unknown	134	2.3	(1.9 - 2.6)
**Individual education (years completed)**	0 to 5	2845	48.0	(46.2 - 49.7)
	6 to 7	672	11.3	(10.5 - 12.2)
	8 to 9	620	10.5	(9.6 - 11.3)
	10 to 11	638	10.8	(9.9 - 11.6)
	>11	921	15.5	(14.5 - 16.5)
	Unknown	233	3.9	(3.4 - 4.4)
**Household SEP quintile** ^**3**^	1	1142	19.3	(18.1 - 20.4)
	2	1249	21.1	(19.9 - 22.2)
	3	1141	19.2	(18.1 - 20.4)
	4	1205	20.3	(19.2 - 21.5)
	5	1128	19.0	(17.9 - 20.1)
	Unknown	64	1.1	(0.8 - 1.3)
**Road distance to nearest health centre or clinic (km)**	0 - 1	1925	32.5	(31.0 - 33.9)
	1 - 3	1497	25.2	(24.0 - 26.5)
	>3	2507	42.3	(40.6 - 43.9)
**Illness duration (months)**	0 to 1	1923	32.4	(31.0 - 33.9)
	>1 to 2	621	10.5	(9.7 - 11.3)
	>2 to 6	1195	20.2	(19.0 - 21.3)
	>6 to 24	1142	19.3	(18.1 - 20.4)
	>24	279	4.7	(4.2 - 5.3)
	Unknown	769	13.0	(12.1 - 13.9)
**Prior mortality rate in residence (deaths/1000 PYO)**	0	2872	48.4	(46.7 - 50.2)
	>0 - 10	1243	21.0	(19.8 - 22.1)
	>10 - 20	985	16.6	(15.6 - 17.7)
	>20	829	14.0	(13.0 - 14.9)
**Cause of death**	HIV/AIDS and TB	2833	47.8	(46.0 - 49.5)
	Other	2409	40.6	(39.0 - 42.3)
	External	362	6.1	(5.5 - 6.7)
	Unknown	325	5.5	(4.9 - 6.1)

^1^The study population comprises adults (aged over 18) who died in the Agincourt sub-district between 2003 and 2011 for whom healthcare utilisation data was available.

^2^South Africa indicates those born in South Africa, Mozambique represents those who either in-migrated from Mozambique or were the children of fathers in-migrating from Mozambique.

^3^SEP (socioeconomic position) quintiles are a measure of the level of asset ownership in a household. Quintile 1 includes the households with the lowest levels of asset ownership and quintile 5 those with the highest levels.

**Figure 2:**
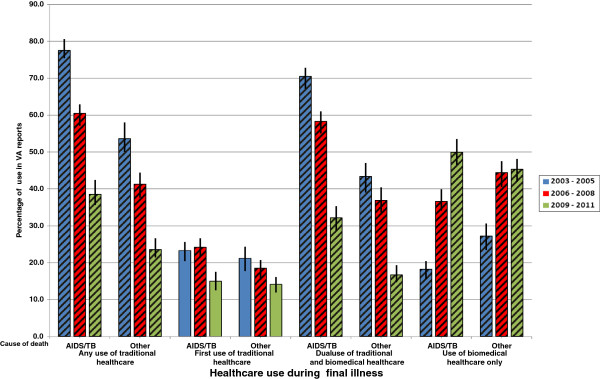
**Short title – Patterns of healthcare use amongst those dying of HIV/AIDS and TB in the study population between 2003 and 2011 stratified by cause of death.** Legend – This plot shows the percentage of the study population dying in each 3 year period for which a particular category of traditional and/or biomedical healthcare use was reported in the verbal autopsy interview. The study population comprises adults (aged over 18) who died in the Agincourt sub-district between 2003 and 2011 for whom healthcare utilisation data was available. Vertical lines indicate the 95% confidence intervals. Diagonal shading indicates that the p-value for the difference in percentages in the type of healthcare usage between the two cause of death categories in a particular time period was less than 0.05. Full results tables are shown in the additional materials (Additional file 2: Tables S2-S5).

An assessment was made of the effect of excluding individuals from the study population due to missing HCU data (Additional file 2: Table S1). The entire population of deceased had a higher percentage of deaths of unknown cause than the study population (p < 0.001) and the distribution of illness duration categories differed between the two groups (p < 0.001) although there was no evidence for a difference in the mean values of illness duration. For those dying of HIV/AIDS and TB, reported TH use decreased from 77.5% to 38.5% between 2003–2005 and 2009–2011, whilst for those dying of other causes the decrease was from 53.6% to 23.6% (Figure 2 & Additional file 2: Table S2). The level of TH use for those dying of HIV/AIDS and TB was significantly higher than that for those dying of other causes in all three time periods (p < 0.001 in each case). A decrease in the percentage of deaths for which TH use was the first treatment option was seen for HIV/AIDS and TB and other causes of death between 2006–2008 and 2009–2011. First use of TH was significantly higher for those dying of HIV/AIDS and TB than for those dying of other causes in 2006–2008 but not in 2009-2011(Figure 2 & Additional file 2: Table S3).The level of reported dual use of biomedical treatment and TH was significantly higher for those dying of HIV/AIDS and TB than for those dying of other causes in each time period. In both CoD categories there were large decreases in the percentage using both biomedical treatments and traditional and herbal medicines between each successive time period (Figure 2 & Additional file 2: Table S4). The percentage of deaths for which only biomedical treatment was reported was higher for those dying of other causes than those dying of HIV/AIDS and TB in 2003 to 2005 (27.2% vs. 18.2% ) and 2006 to 2008 (44.4% vs. 36.6%) (Figure 2 and Additional file 2: Table S5). However in 2009 to 2011 sole use of biomedical treatment was reported for 49.9% of HIV/AIDS and TB deaths and 45.3% of deaths due to other causes, the difference between the two was significant (p = 0.035).

A bivariate logistic regression analysis was carried out in order to assess which variables were individually associated with the level of TH use amongst those dying of HIV/AIDS and TB (Table 2). Higher TH use was seen for those with: Mozambican origin (compared to South Africans), lower levels of education, homes 1–3 km from the nearest clinic (compared to those living within 1 km), longer illness duration and a prior mortality rate in their dwelling of 10–20 deaths/1000 PYO (compared to no deaths). After controlling for the effect of other variables in a multivariate analysis it was found that Mozambicans used TH more than South Africans, there was a significant decrease in TH use in each subsequent 3-year period and the level of TH use increased with increasing duration of illness. For the other variables included in the model, the significance seen in the bivariate analysis was lost.

**Table 2:** **Bivariate and multivariate logistic regression analyses of determinants of any traditional healthcare use amongst those dying of HIV/AIDS in the study population** ^**1**^

				Bivariate analysis		Multivariate analysis	
Characteristics	Category	Total	N (%)	Odds ratio	95% CI (p value)	Odds ratio	95% CI (p value)
**Gender**	Female	1430	879 (61.47)	1 (ref)			
	Male	1403	828 (59.02)	0.90	0.77 - 1.05 (0.198)		
**Age at death (years)**	18 - 49	1974	1194 (60.49)	1 (ref)			
	50 – 65	533	325 (60.98)	1.01	0.83 - 1.24 (0.903)		
	>65	326	188 (57.67)	0.88	0.69 - 1.12 (0.304)		
**Country of origin** ^**2**^	Mozambique	936	602 (64.32)	1 (ref)		1(ref)	
	South Africa	1894	1104 (58.29)	0.77	0.65 - 0.91 (0.002)	0.72	0.60 - 0.87 (0.001)
**Year of death**	2003 - 2005	979	759 (77.53)	1 (ref)		1(ref)	
	2006 - 2008	1067	645 (60.45)	0.44	0.36 - 0.54 (<0.001)	0.50	0.40 - 0.61 (<0.001)
	2009 - 2011	787	303 (38.50)	0.18	0.15 - 0.22 (<0.001)	0.23	0.18 - 0.29 (<0.001)
**Residence status**	Temporary	801	476 (59.43)	1 (ref)			
	Permanent	1949	1187 (60.90)	1.06	0.89 - 1.26 (0.529)		
**Individual education (years completed)**	0 to 5	1165	744 (63.86)	1 (ref)		1(ref)	
	6 to 7	361	217 (60.11)	0.84	0.66 - 1.08 (0.178)	1.06	0.81 - 1.38 (0.669)
	8 to 9	332	208 (62.65)	0.94	0.72 - 1.21 (0.62)	1.24	0.93 - 1.64 (0.138)
	10 to 11	360	206 (57.22)	0.75	0.59 - 0.96 (0.024)	1.10	0.84 - 1.45 (0.482)
	>11	486	262 (53.91)	0.67	0.54 - 0.84 (<0.001)	0.93	0.73 - 1.19 (0.572)
**Household SEP quintile** ^**3**^	1	602	376 (62.46)	1 (ref)			
	2	643	398 (61.90)	0.97	0.77 - 1.23 (0.815)		
	3	533	320 (60.04)	0.91	0.71 - 1.16 (0.452)		
	4	554	320 (57.76)	0.84	0.66 - 1.07 (0.152)		
	5	473	279 (58.99)	0.86	0.67 - 1.11 (0.262)		
**Road distance to nearest health centre or clinic (km)**	0 - 1	907	538 (59.32)	1 (ref)		1(ref)	
	1 - 3	730	466 (63.84)	1.22	0.99 - 1.50 (0.058)	1.14	0.91 - 1.42 (0.253)
	>3	1196	703 (58.78)	0.98	0.81 - 1.17 (0.787)	1.03	0.84 - 1.25 (0.795)
**Illness duration (months)**	0 to 1	419	164 (39.14)	1 (ref)		1(ref)	
	>1 to 2	401	193 (48.13)	1.43	1.07 - 1.91 (0.014)	1.31	0.97 - 1.77 (0.081)
	>2 to 6	798	480 (60.15)	2.37	1.84 - 3.04 (<0.001)	2.16	1.66 - 2.80 (<0.001)
	>6 to 24	847	620 (73.20)	4.24	3.28 - 5.49 (<0.001)	2.84	2.16 - 3.73 (<0.001)
	>24	191	151 (79.06)	5.85	3.88 - 8.80 (<0.001)	2.79	1.81 - 4.32 (<0.001)
**Prior Mortality rate in residence (deaths/1000 PYO)**	0	1329	777 (58.47)	1 (ref)		1(ref)	
	>0 - 10	568	347 (61.09)	1.10	0.89 - 1.35 (0.374)	1.06	0.84 - 1.32 (0.634)
	>10 - 20	478	305 (63.81)	1.27	1.02 - 1.59 (0.034)	1.13	0.89 - 1.44 (0.304)
	>20	458	278 (60.70)	1.09	0.87 - 1.36 (0.472)	1.01	0.79 - 1.28 (0.956)

^1^The study population comprises adults (aged over 18) who died in the Agincourt sub-district between 2003 and 2011 for whom healthcare utilisation data was available.

^2^South Africa indicates those born in South Africa, Mozambique represents those who either inmigrated from Mozambique or were the children of fathers in-migrating from Mozambique.

^3^SEP (socioeconomic position) quintiles are a measure of the level of asset ownership in a household. Quintile 1 includes the households with the lowest levels of asset ownership and quintile 5 those with the highest levels.

In 2003–2005 the level of TH use was higher for those of Mozambican origin than for South Africans. By 2006–2008 TH use remained higher for Mozambicans but there was evidence from the interaction term (Table 3) that the difference had decreased. (Odds Ratio between the two periods 1.48 95% CI 0.94 – 2.32 p 0.089). In 2003–2005 TH use was lower for those who had completed secondary education than for those with less than 5 years of education. Again there was evidence from the interaction term (Table 3) that by 2009–2011 this difference had decreased (Odds Ratio 1.71 95% CI 0.92 – 3.17 p 0.088).

**Table 3:** **Interaction terms included** ^**1**^ **in the multivariate logistic regression model of traditional healthcare use amongst the study population** ^**2**^

Determinant	Comparison	Period	Odds ratio	95% Confidence interval p value
**Country of origin** ^**3**^	(SA/Moz)	2003-2005	1 (ref)	
	(SA/Moz)	2006-2008	1.48	0.94 - 2.32 (0.089)
	(SA/Moz)	2009-2011	1.36	0.84 - 2.18 (0.209)
**Education status (years of education completed)**	6 - 7/0 - 5	2003-2005	1 (ref)	
	6 - 7/0 - 5	2006-2008	1.05	0.54 - 2.02 (0.887)
	6 - 7/0 - 5	2009-2011	0.97	0.48 - 1.98 (0.944)
	8 - 9/0 - 5	2003-2005	1 (ref)	
	8 - 9/0 - 5	2006-2008	0.79	0.39 - 1.58 (0.501)
	8 - 9/0 - 5	2009-2011	0.92	0.44 - 1.92 (0.825)
	10 - 11/0 - 5	2003-2005	1 (ref)	
	10 - 11/0 - 5	2006-2008	0.73	0.36 - 1.49 (0.389)
	10 - 11/0 - 5	2009-2011	0.63	0.31 - 1.29 (0.209)
	>11/0 - 5	2003-2005	1 (ref)	
	>11/0 - 5	2006-2008	1.10	0.62 - 1.95 (0.743)
	>11/0 - 5	2009-2011	1.71	0.92 - 3.17 (0.088)

^1^Interaction terms were included when a p value < 0.1 was seen for at least one level of the interaction.

^2^The study population comprised adults (aged over 18) who died of HIV/AIDS related conditions or tuberculosis in the Agincourt sub-district between 2003 and 2011 for whom healthcare utilisation data was available.

^3^SA represents those born in South Africa, Moz represents those who either in-migrated from Mozambique or were the children of fathers in-migrating from Mozambique.

A subsequent analysis in which the CoD likelihoods were not used as weights in the regression made only small numerical differences to the odds ratios and no differences to the associations.

## Discussion

We carried out an analysis comparing the prevalence of traditional healthcare (TH) use between those dying of HIV/AIDS-related disease and pulmonary tuberculosis (HIV/AIDS and TB) and those dying of other causes. The study was located in a rural area of South Africa and analysed data on deaths occurring between 2003 and 2011 a period of time when antiretroviral therapy (ART) became available within the study area. We also sought to identify determinants predictive of TH use amongst those dying of HIV/AIDS and TB in this population.

Over the period of the study, reported TH use decreased by approximately 50% amongst both those dying of HIV/AIDS and TB and those dying of other causes. The level of TH use amongst those dying of HIV/AIDS and TB was significantly higher than that for those dying of other causes throughout the period of the study. Similar decreases in concomitant use of TH and biomedical treatment during the final illness were also seen. Decreases in the number reporting that TH was sought prior to biomedical treatment were also seen in each cause of death (CoD) category. There was some evidence of the decrease occurring later for those dying of HIV/AIDS and TB than for those dying of other causes. In 2003–2005, the percentage reporting that only biomedical treatment was used during the deceased’s final illness was lower amongst those dying of HIV/AIDS and TB than those dying of other causes. By 2009–2011 this had reversed and the level of sole use of biomedical treatment was higher for HIV/AIDS and TB deaths. Higher levels of reported TH use were found to be associated with an earlier year of death, longer duration of the illness and Mozambican origin in multivariate logistic regression models in which the effect of other variables was controlled for.

In this study no associations were identified between higher levels of TH use amongst those dying of HIV/AIDS and TB and various socio-demographic characteristics such as age, gender, education level and socio-economic position (SEP). In contrast Sato and colleagues surveying traditional medicine use in Ghana identified an association between rising income and an increased use of biomedical treatment whereas decreasing income was associated with a greater use of traditional medicine [38]. Two previous South African studies [27, 34] found that lower wealth, as indicated by grant receipt or lower asset ownership were associated with increased use of TH. The fact that in the study area ART was available at no cost from 2007 onwards may explain these differences. It has also been reported that patterns of TH use appear to vary widely across the country [9] which may be another explanation for the differences.

There was evidence from the interaction terms added to the multivariate model that the relative differences between South Africans and Mozambicans in the levels of TH use for those dying of HIV/AIDS and TB decreased between 2003–2005 and 2006–2008. Similarly when considering the effect of education the models indicated that the relative differences in levels of TH use between those who had completed their secondary level of education and those who had not completed primary education decreased between 2006–2008 and 2009–2011. This would imply that as the overall level of TH use decreased, educational attainment and country of origin were no longer important determinants of TH use in this population.

A strength of this study is that data on healthcare use patterns were collected consistently prior to and during the period of the ART roll-out. Previous studies [1, 3, 9, 21, 33, 35–37] being cross-sectional are not able to reflect this changing temporal dynamic. Also as we were able to assign causality to each death in an objective manner, we were able to assess whether changing use patterns were disease specific or more general.

A limitation with our study is that the data set comprised only individuals who had died. This was a subset of all of the sick who either did not receive treatment or for whom the treatment was unsuccessful. It is difficult to assess the direction of bias when these results are compared to population surveys of healthcare use amongst the living. As an individual is likely to have much higher levels of use of biomedical or traditional healthcare in the final stages of their illness, this may be representative of changes in the whole population; however, further studies are needed to confirm this. As the reports of healthcare use come from care-givers of the deceased, these may be inaccurate due to recall bias and a possible lack of involvement in the deceased’s healthcare in the early stages of the illness. It may also be that a perception amongst the respondents of the lack of approval by biomedical practitioners and researchers made them less likely to report TH use. However, our use of fieldworkers who are members of the same community as the respondents who were specifically trained to deal with confidential and sensitive data should lessen the risk of inaccurate data being given. Also as data was collected from a care-giver rather than the individual themselves the respondent could not personally be stigmatised due to information given. The data was collected prior to a cause of death being assigned so neither the fieldworkers or the caregiver would know the death was assigned as due to HIV/AIDS at the time of the interview, we are not aware of stigmatisation associated with consulting traditional practitioners for medical conditions other than HIV/AIDS. As we do not have data on the specific types of treatment provided by either the traditional or biomedical practitioners we cannot draw any firm conclusions about the level of concomitant ART and herbal medicine use. Also due to specific cultural and social factors different patterns and determinants of use may occur in other areas of rural South Africa and sub-Saharan Africa.

Following from the ideas on how healthcare choices are made in this community, as put forward by Golooba-Mutebi and Tollman in 2002 [28], it may be hypothesised that the decrease in TH use was a response to personal, family or community experience of the effectiveness of ART. This may be coupled with a lessening in associated stigma and greater acceptance of HIV-related illness in these communities.

## Conclusions

In conclusion, prior to the availability of ART, traditional herbal or spiritual therapies were the only healthcare options available in this community for those with HIV-related disease. With the decentralised ART roll-out and improved access to treatment there was a decrease in HIV-associated mortality and a decrease in TH use. The persisting high levels of concomitant use of traditional and biomedical treatment for HIV-related disease are a potential cause for concern amongst biomedical practitioners due to the possibility of antagonistic interactions between ART drugs and the traditional herbal treatments. A recent Mozambican study indicated a willingness on the part of traditional practitioners to engage with allopathic providers, but noted that the success of previous initiatives to achieve this had been limited [56]. A review of projects across sub-Saharan Africa to integrate traditional medical practitioners with biomedical providers has shown that the sectors can work effectively together but highlighted the need for more effective evaluation of such initiatives in order to build an evidence base on best practice for such projects [5].

Based on our findings, both regular repeated cross-sectional analyses of healthcare use and in-depth qualitative studies of the rapidly changing patterns of healthcare would be useful to enable us to gain a better understanding of changes in health seeking behavior in this community in response to the developing HIV pandemic and the availability of ART.

## Electronic supplementary material

Additional file 1: **Agincourt verbal autopsy health care utilization instrument.** This form was used to collect the data on the use made of different types of healthcare by the deceased person during the final stages of the illness. (PDF 63 KB)

Additional file 2: Table S1: Comparison of descriptive variables for the entire population (those dying between 2003 and 2011 in the Agincourt HDSS) and the study population (the subset of the entire population for whom healthcare utilisation data was available). **Tables S2–S5** comparisons of the percentages of those dying between 2003 and 2011 for different categories of biomedical and traditional healthcare use. The categories used are as follows. **Table S2.** Any use of traditional or herbal treatment during final illness. **Table S3.** First use of traditional or herbal treatment in the healthcare seeking pathway during the final illness. **Table S4.** Use of both traditional or herbal treatments and biomedical treatments during the final illness. **Table S5.** Sole use of biomedical treatment during the final illness. (PDF 404 KB)

## Abbreviations

ART: Antiretroviral treatment
CoD: Cause of death
CI: Confidence interval
HCU: Healthcare utilisation
HDSS: Health and socio-demographic surveillance system
HIV: Human immunodeficiency virus
HIV/AIDS and TB: Human immunodeficiency virus -related disease and pulmonary tuberculosis
OR: Odds ratio
PYO: Person-years of observation
SEP: Socio-economic position
TB: Tuberculosis
TH: Traditional healthcare
THM: Traditional herbal medicines
TMP: Traditional medical practitioner
USD: US dollar
VA: Verbal autopsy
VCT: Voluntary counseling and testing
WHO: World Health Organization.
