# Supplementary material for: Changing use of traditional healthcare amongst those dying of HIV related disease and TB in rural South Africa from 2003 – 2011: a retrospective cohort study
Source: BMC Complement Altern Med. 2014 Dec 17;14:504. doi: 10.1186/1472-6882-14-504 (PMC4325963; doi:10.1186/1472-6882-14-504)
Supplement: Supplementary file 1 — Additional file 1: Agincourt verbal autopsy health care utilization instrument. This form was used to collect the data on the use made of different types of healthcare by the deceased person during the final stages of the illness. (PDF 63 KB) [file 12906_2014_2096_MOESM1_ESM.pdf]

Health Care Utilisation During Terminal Illness

Res status	If M, ill in site? Y/N	Immunisation: child under 5 years old (only if RTH card available) YELLOW / WHITE									If no RTH card, was child immunised?	Did deceased have access to medical aid or employer support for health care?	What action was taken during the deceased's terminal illness? (out-patient care only)					If action codes 1, 2, 3, 4, 11 and never admitted to hospital, why was western care not consulted? (can mark > 1 code)	Was deceased admitted to hospital during terminal illness? Y/N/X	If yes, number of admissions in past year?	Which hospital (allow more than one code)
													1st	2nd	3rd	4th	Any > 4 actions? Y				
		BCG	OPV Polio	DPT	Hib	HepB	Measles	RV	DTaP-IPVHib	PCV	Y/N/X		Action codes below					Why no action codes below			

Immunisation
Number of doses:
0, 1, 2, 3, 4

Medical aid / Employer support?
0=no
1=covered by a medical aid for whenever person needs to go to doctor etc
2=is covered by health insurance for a specific disease(s)
3=is allowed to go to employer's clinic/hospital
4=access to free public HOSPITAL care
5=don't know

Action codes (for out-patient care):
1. None over entire illness period
2. Took natural home remedy (eg herbs, ash, roots etc)
3. Took left-over medication from doctor or clinic
4. Drugs from a shop
5. Private chemist
6. Public clinic / health center
7. Public doctor didn't have to pay for (district surgeon), not in a public hospital
8. Private doctor (had to pay or on medical aid)
9. Out-patient care by **private specialist** doctor (referred to by another doctor / specialises in certain illnesses) that had to pay for?
10. Out-patient care by **public specialist** doctor (referred to by another doctor / specialises in certain illnesses) that did not have to pay for?
11. Faith healer or Traditional healer (sangoma, inyanga)
12. Out-patient or casualty in public hospital
13. Out-patient or casualty in private hospital
14. Second public clinic / health centre
15. Don't know
16. Other (specify)

Why no action/Why no western care for this illness?
1. Did not seem sick enough
2. Thought illness would get better on its own
3. Sudden death, no time for treatment
4. Did not know what to do
5. No time, too busy
6. Not enough money for consultation
7. Transport too expensive
8. Too far to travel
9. Needed permission of another person eg husband/father/mother etc, and not available/refused
10. Patient refused to go for care
11. Nobody to go with him/her
12. Nobody to care for children
13. No drugs at health care facility
14. Staff attitudes poor
15. Not enough privacy
16. Too little time with health worker
17. Queue too long, wait too long
18. Western care could do nothing for illness
19. Other (specify)
20. Don't' know

Hospitalisation, which one?
List codes for ALL hospital admissions in past year. If person was admitted to one hospital and then referred from that hospital to a second hospital, list both codes and bracket them together, eg (3,7). If person was admitted to two hospitals but at different times, list both codes but do not bracket together, eg 3,7.

Yes,….Which one?
1= Mapulaneng
2= Matikwane
3= Tintswalo
4= Rob Ferreira
5= Nelspruit Private Hospital
6= Pietersburg Mankweng
7=Garankuwa
8=Bhubezi
9= Other (write name)
10=Don't know

DISPLAY
