# Supplementary material for: Changing use of traditional healthcare amongst those dying of HIV related disease and TB in rural South Africa from 2003 – 2011: a retrospective cohort study
Source: BMC Complement Altern Med. 2014 Dec 17;14:504. doi: 10.1186/1472-6882-14-504 (PMC4325963; doi:10.1186/1472-6882-14-504)
Supplement: Supplementary file 2 — Additional file 2: Table S1: Comparison of descriptive variables for the entire population (those dying between 2003 and 2011 in the Agincourt HDSS) and the study population (the subset of the entire population for whom healthcare utilisation data was available). Tables S2–S5 comparisons of the percentages of those dying between 2003 and 2011 for different categories of biomedical and traditional healthcare use. The categories used are as follows. Table S2. Any use of traditional or herbal treatment during final illness. Table S3. First use of traditional or herbal treatment in the healthcare seeking pathway during the final illness. Table S4. Use of both traditional or herbal treatments and biomedical treatments during the final illness. Table S5. Sole use of biomedical treatment during the final illness. (PDF 404 KB) [file 12906_2014_2096_MOESM2_ESM.pdf]

	Entire Population				Study Population [1]								
**Characteristic**	**n**	**%**	**(95% CI)**	**Mean**	**n**	**%**	**(95% CI)**	**Mean**	**n (Expected)**	**Pearson chitest p value**	**ttest absolute difference between means**	**ttest sample mean < population mean**	**ttest sample mean > population mean**
**TOTAL**	**6392**	**100.00**			**5929**	**100.00**							
**Gender**													
Female	**3047**	**47.67**	**(45.98 - 49.36)**		**2831**	**47.75**	**(45.99 - 49.51)**		2826	**0.897**			
Male	**3345**	**52.33**	**(50.56 - 54.10)**		**3098**	**52.25**	**(50.41 - 54.09)**		3103				
**Age at Death (years)**													
18 to 49	**3571**	**55.87**	**(54.03 - 57.70)**	**49.76**	**3283**	**55.37**	**(53.48 - 57.27)**	**50.04**	3312	**0.703**	**0.279**	**0.861**	**0.139**
50 to 65	**1253**	**19.60**	**(18.52 - 20.69)**		**1167**	**19.68**	**(18.55 - 20.81)**		1162				
> 65	**1568**	**24.53**	**(23.32 - 25.74)**		**1479**	**24.95**	**(23.67 - 26.22)**		1454				
**Country of Origin [2]**													
Mozambique	**1929**	**30.18**	**(28.83 - 31.53)**		**1823**	**30.75**	**(29.34 - 32.16)**		1789	**0.629**			
South Africa	**4458**	**69.74**	**(67.70 - 71.79)**		**4101**	**69.17**	**(67.05 - 71.29)**		4135				
Unknown	**5**	**0.08**	**(0.01 - 0.15)**		**5**	**0.08**	**(0.01 - 0.16)**		5				
**Year of Death**													
2003 to 2005	**1994**	**31.20**	**(29.83 - 32.56)**		**1781**	**30.04**	**(28.64 - 31.43)**		1850	**0.094**			
2006 to 2008	**2276**	**35.61**	**(34.14 - 37.07)**		**2115**	**35.67**	**(34.15 - 37.19)**		2111				
2009 to 2011	**2122**	**33.20**	**(31.79 - 34.61)**		**2033**	**34.29**	**(32.80 - 35.78)**		1968				
**Residence Status**													
Temporary	**1829**	**28.61**	**(27.30 - 29.93)**		**1629**	**27.48**	**(26.14 - 28.81)**		1697	**0.103**			
Permanent	**4410**	**68.99**	**(66.96 - 71.03)**		**4166**	**70.26**	**(68.13 - 72.40)**		4091				
Unknown	**153**	**2.39**	**(2.01 - 2.77)**		**134**	**2.26**	**(1.88 - 2.64)**		142				
**Individual Education (years completed)**													
0 to 5	**3026**	**47.34**	**(45.65 - 49.03)**	**5.36**	**2845**	**47.98**	**(46.22 - 49.75)**	**5.3**	2807	**0.934**	**0.372**	**0.186**	**0.814**
6 to 7	**733**	**11.47**	**(10.64 - 12.30)**		**672**	**11.33**	**(10.48 - 12.19)**		680				
8 to 9	**667**	**10.43**	**(9.64 - 11.23)**		**620**	**10.46**	**(9.63 - 11.28)**		619				
10 to 11	**706**	**11.05**	**(10.23 - 11.86)**		**638**	**10.76**	**(9.93 - 11.60)**		655				
> 11	**1001**	**15.66**	**(14.69 - 16.63)**		**921**	**15.53**	**(14.53 - 16.54)**		928				
Unknown	**259**	**4.05**	**(3.56 - 4.55)**		**233**	**3.93**	**(3.43 - 4.43)**		240				

Table S1 - Comparison of descriptive variables between the entire population of those dying between 2003 and 2011 in the Agincourt HDSS and the study population [1]

[1] The study population comprises adults (aged over 18) who died in the Agincourt sub-district between 2003 and 2011 for whom healthcare utilisation data was available.

[2] South Africa indicates those born in South Africa, Mozambique represents those who either in-migrated from Mozambique or were the children of fathers in-migrating from Mozambique.

[3] SEP (socioeconomic position) quintiles are a measure of the level of asset ownership in a household. Quintile 1 includes the households with the lowest levels of asset ownership and quintile 5 those with the highest levels.

	Entire Population				Study Population [1]								
Characteristic	n	%	(95% CI)	Mean	n	%	(95% CI)	Mean	n (Expected)	Pearson chitest p value	ttest absolute difference between means	ttest sample mean < population mean	ttest sample mean > population mean
**Household SEP quintile [3]**													
1	**1230**	**19.24**	**(18.17 - 20.32)**		**1142**	**19.26**	**(18.14 - 20.38)**		1141	**0.992**			
2	**1347**	**21.07**	**(19.95 - 22.20)**		**1249**	**21.07**	**(19.90 - 22.23)**		1249				
3	**1220**	**19.09**	**(18.02 - 20.16)**		**1141**	**19.24**	**(18.13 - 20.36)**		1132				
4	**1296**	**20.28**	**(19.17 - 21.38)**		**1205**	**20.32**	**(19.18 - 21.47)**		1202				
5	**1225**	**19.16**	**(18.09 - 20.24)**		**1128**	**19.03**	**(17.91 - 20.14)**		1136				
Unknown	**74**	**1.16**	**(0.89 - 1.42)**		**64**	**1.08**	**(0.81 - 1.34)**		69				
**Distance to Nearest Clinic (km)**													
0 - 1	**2053**	**32.12**	**(30.73 - 33.51)**	**3.03**	**1925**	**32.47**	**(31.02 - 33.92)**	**3.05**	1904	**0.735**	**0.641**	**0.680**	**0.320**
1 - 3	**1640**	**25.66**	**(24.42 - 26.90)**		**1497**	**25.25**	**(23.97 - 26.53)**		1521				
> 3	**2699**	**42.22**	**(40.63 - 43.82)**		**2507**	**42.28**	**(40.63 - 43.94)**		2503				
**Illness Duration (months)**													
0 to 1	**1934**	**30.26**	**(28.91 - 31.61)**	**8.06**	**1923**	**32.43**	**(30.98 - 33.88)**	**8.05**	1794	**< 0.001**	**0.980**	**0.490**	**0.510**
> 1 to 2	**625**	**9.78**	**(9.01 - 10.54)**		**621**	**10.47**	**(9.65 - 11.30)**		580				
> 2 to 6	**1208**	**18.90**	**(17.83 - 19.96)**		**1195**	**20.16**	**(19.01 - 21.30)**		1120				
> 6 to 24	**1152**	**18.02**	**(16.98 - 19.06)**		**1142**	**19.26**	**(18.14 - 20.38)**		1069				
> 24	**283**	**4.43**	**(3.91 - 4.94)**		**279**	**4.71**	**(4.15 - 5.26)**		263				
Unknown	**1190**	**18.62**	**(17.56 - 19.67)**		**769**	**12.97**	**(12.05 - 13.89)**		1104				
**Previous Mortality Rate at Residence (deaths/1000 PY)**													
0	**3093**	**48.39**	**(46.68 - 50.09)**	**10.12**	**2872**	**48.44**	**(46.67 - 50.21)**	**9.85**	2869	**0.905**	**0.378**	**0.188**	**0.812**
> 0 - 10	**1320**	**20.65**	**(19.54 - 21.76)**		**1243**	**20.96**	**(19.80 - 22.13)**		1224				
> 10 - 20	**1071**	**16.76**	**(15.75 - 17.76)**		**985**	**16.61**	**(15.58 - 17.65)**		993				
> 20	**908**	**14.21**	**(13.28 - 15.13)**		**829**	**13.98**	**(13.03 - 14.93)**		842				
**Cause of Death**													
AIDS/TB	**2930**	**45.84**	**(44.18 - 47.50)**		**2833**	**47.78**	**(46.02 - 49.54)**		2718	**< 0.001**			
Other	**2430**	**38.02**	**(36.50 - 39.53)**		**2409**	**40.63**	**(39.01 - 42.25)**		2254				
External	**378**	**5.91**	**(5.32 - 6.51)**		**362**	**6.11**	**(5.48 - 6.73)**		351				
Unknown	**654**	**10.23**	**(9.45 - 11.02)**		**325**	**5.48**	**(4.89 - 6.08)**		607				

Table S1 - Comparison of descriptive variables between the entire population of those dying between 2003 and 2011 in the Agincourt HDSS and the study population [1]

[1] The study population comprises adults (aged over 18) who died in the Agincourt sub-district between 2003 and 2011 for whom healthcare utilisation data was available.

[2] South Africa indicates those born in South Africa, Mozambique represents those who either in-migrated from Mozambique or were the children of fathers in-migrating from Mozambique.

[3] SEP (socioeconomic position) quintiles are a measure of the level of asset ownership in a household. Quintile 1 includes the households with the lowest levels of asset ownership and quintile 5 those with the highest levels.

	HIV/TB				Other Causes				
**Year of Death**	**Any use of traditional or herbal remedies (N)**	**Total**	**%**	**95 % CI**	**Any use of traditional or herbal remedies (N)**	**Total**	**%**	**95 % CI**	**p value (difference between percentages)**
**2003 to 2005**	759	979	77.5	(75.4 - 80.6)	319	595	53.6	(50.0 - 58.0)	< 0.001
**2006 to 2008**	645	1067	60.4	(57.1 - 62.9)	328	795	41.3	(37.6 - 44.4)	<0.001
**2009 to 2011**	303	787	38.5	(35.6 - 42.4)	240	1019	23.6	(21.4 - 26.6)	<0.001
**Totals**	**1707**	**2833**	**60.3**	**(58.2 - 61.8)**	**887**	**2409**	**36.8**	**(35.1 - 38.9)**	**<0.001**

Table S2 - Percentages of adults (aged over 18) who died in the Agincourt sub-district between 2003 and 2011 for whom healthcare utilisation data was available making any use of traditional or herbal treatment during the final illness

	HIV/TB				Other Causes				
**Year of Death**	**Use of traditional or herbal remedies as the first source of healthcare**	**Total**	**%**	**95 % CI**	**Use of traditional or herbal remedies as the first source of healthcare**	**Total**	**%**	**95 % CI**	**p value (difference between percentages)**
**2003 to 2005**	228	979	23.3	(20.4 - 25.6)	126	595	21.2	(17.7 - 24.3)	0.355
**2006 to 2008**	258	1067	24.2	(21.4 - 26.6)	147	795	18.5	(15.3 - 20.7)	0.002
**2009 to 2011**	118	787	15.0	(12.5 - 17.5)	144	1019	14.1	(11.9 - 16.1)	0.549
**Totals**	**604**	**2833**	**21.3**	**(19.5 - 22.5)**	**417**	**2409**	**17.3**	**(15.5 - 18.5)**	**<0.001**

Table S3 - Percentages of adults (aged over 18) who died in the Agincourt sub-district between 2003 and 2011 for whom healthcare utilisation data was available using traditional or herbal treatment as the first source of healthcare for the final illness

	HIV/TB				Other Causes				
**Year of Death**	**Use of both traditional and biomedical treatments**	**Total**	**%**	**95 % CI**	**Use of both traditional and biomedical treatments**	**Total**	**%**	**95 % CI**	**p value (difference between percentages)**
**2003 to 2005**	690	979	70.5	(67.1 - 72.8)	258	595	43.4	(39.0 - 47.0)	<0.001
**2006 to 2008**	622	1067	58.3	(55.0 - 61.0)	293	795	36.9	(33.6 - 40.4)	<0.001
**2009 to 2011**	253	787	32.1	(28.7 - 35.3)	170	1019	16.7	(14.7 - 19.3)	<0.001
**Totals**	**1565**	**2833**	**55.2**	**(53.2 - 56.8)**	**721**	**2409**	**29.9**	**(28.2 - 31.8)**	**<0.001**

Table S4 - Percentages of adults (aged over 18) who died in the Agincourt sub-district between 2003 and 2011 for whom healthcare utilisation data was available using traditional or herbal and biomedical treatments during the final illness

	HIV/TB				Other Causes				
**Year of Death**	**Use of only biomedical treatments**	**Total**	**%**	**95 % CI**	**Use of only biomedical treatments**	**Total**	**%**	**95 % CI**	**p value (difference between percentages)**
**2003 to 2005**	178	979	18.2	(15.5 - 20.4)	162	595	27.2	(23.4 - 30.6)	< 0.001
**2006 to 2008**	391	1067	36.6	(34.1 - 39.9)	353	795	44.4	(40.5 - 47.5)	0.002
**2009 to 2011**	393	787	49.9	(46.5 - 53.5)	462	1019	45.3	(41.9 - 48.1)	0.035
**Totals**	**962**	**2833**	**34.0**	**(32.3 - 35.7)**	**977**	**2409**	**40.6**	**(39.0 - 43.0)**	**<0.001**

Table S5 - Percentages of adults (aged over 18) who died in the Agincourt sub-district between 2003 and 2011 for whom healthcare utilisation data was available using only biomedical treatments during the final illness
